# Ability of Surface Electrocardiography in Predicting Ventricular Arrhythmias in Dogs with Secondary Atrial Fibrillation

**DOI:** 10.3390/ani15203057

**Published:** 2025-10-21

**Authors:** Giovanni Romito, Chiara Mazzoldi, Carlotta Valente, Helen Poser, Giulia Arcuri, Barbara Contiero, Carlo Guglielmini

**Affiliations:** 1 Department of Veterinary Medical Sciences, Alma Mater Studiorum-Università di Bologna, Via Tolara di Sopra 50, 40064 Ozzano Emilia, Italy; giovanni.romito2@unibo.it (G.R.); chiara.mazzoldi2@unibo.it (C.M.); 2 Department of Animal Medicine, Productions and Health (MAPS), University of Padua, Viale dell’Università 16, 35020 Legnaro, Italy; carlotta.valente@unipd.it (C.V.); helen.poser@unipd.it (H.P.); giulia.arcuri@phd.unipd.it (G.A.); barbara.contiero@unipd.it (B.C.)

**Keywords:** cardiac arrhythmia, echocardiography, electrocardiography, Holter monitoring, supraventricular arrhythmia, ventricular premature complexes

## Abstract

**Simple Summary:**

Atrial fibrillation (AF) and ventricular arrhythmias (VAs) are the most common pathological arrhythmias of dogs and are associated with a poor prognosis in those with cardiac disease. While several studies have investigated the clinical features of AF and VAs individually, few have documented their concurrent occurrence. In this study, we evaluated 35 dogs with AF secondary to cardiac disease to assess whether a routine electrocardiographic (ECG) examination—defined as 2 to 5 min in duration—could detect the presence and severity of concurrent VAs. Continuous 24 h ECG monitoring (Holter) was used as the reference standard. Although all 35 dogs exhibited VAs on Holter monitoring, only 13 (37%) had VAs detectable on routine ECG. A significant positive correlation was observed between the presence of VAs on routine ECG and the severity of VAs identified by Holter monitoring. However, the diagnostic accuracy of routine ECG in predicting VAs deemed severe according to the study’s criteria was only moderate. These findings indicate that while VAs are common in dogs with secondary AF, routine ECG is not a reliable tool for assessing their presence or severity.

**Abstract:**

Atrial fibrillation (AF) and ventricular arrhythmias (VAs) are common pathological arrhythmias of dogs and are both associated with a poor prognosis in those with cardiac disease. This study aimed to assess the ability of 2 to 5 min electrocardiography (routine ECG) to detect the presence and severity of concomitant VAs in dogs with secondary AF. Continuous 24 h ECG monitoring (Holter) was used as the reference standard to identify VAs, quantify the number of premature ventricular ectopic complexes (VPCs) and evaluate the degree of their organization using a modified Lown–Wolf classification scale. In light of the Holter findings, VAs were classified as severe based on two criteria: the presence of more than 100 VPCs and a Lown–Wolf grade ≥ 4. Thirty-five dogs with secondary AF were included, where all exhibited VAs on Holter monitoring. Most dogs had severe VAs, according to both the VPC count (69%) and Lown–Wolf classification (77%). However, only 13 dogs (37%) had VAs detectable on routine ECG. A significant positive correlation was found between the presence of VAs on routine ECG and the severity of VAs identified via Holter. Nevertheless, the diagnostic accuracy of routine ECG in predicting severe VAs was only moderate (68.6% based on VPC count and 60% based on Lown–Wolf grade). Overall, a 2 to 5 min ECG appears to be a highly specific but relatively insensitive tool for detecting VAs in dogs with secondary AF.

## 1. Introduction

Atrial fibrillation (AF) is the most common supraventricular tachyarrhythmia in dogs and is frequently observed in subjects with heart disease leading to atrial enlargement (secondary AF), such as myxomatous mitral valve disease and dilated cardiomyopathy (DCM) [1,2,3,4]. In addition to AF, these cardiac conditions may also be associated with the presence of ventricular arrhythmias (VAs) [5,6,7,8,9,10]. Although some studies have reported the coexistence of both types of arrhythmias, the actual prevalence of VAs in dogs with secondary AF remains to be conclusively defined [8,11]. Identifying VAs in dogs with secondary AF may be clinically relevant, as it may guide the implementation of appropriate antiarrhythmic therapy. In turn, this may reduce arrhythmia-induced mortality, as undetected and untreated VAs may lead to sudden cardiac death, even when AF appears to be well controlled [11,12]. However, the accurate assessment of VAs often requires 24 h Holter monitoring [8,9,10,11], which is not always feasible due to its cost, the need for specific equipment, and technical expertise. Consequently, many dogs with cardiac disease, including those with secondary AF, are assessed only by surface electrocardiogram (ECG), at least at the time of diagnosis. In light of this, the aim of the present study was to evaluate the ability of surface ECG to predict the presence and severity of VAs in dogs with secondary AF.

## 2. Materials and Methods

This retrospective observational study was conducted using clinical data retrieved from the databases of the Veterinary Teaching Hospitals of the Universities of Bologna and Padua. For all dogs included in the study, informed owner consent was obtained, which authorized the diagnostic procedures performed as well as the potential use of clinical data for research purposes.

### 2.1. Animals

Medical records of client-owned dogs diagnosed with secondary AF between February 2014 and September 2024 were retrospectively reviewed from the authors’ databases. To be eligible for inclusion, each dog was required to have (1) a surface ECG recorded for 2 to 5 min (routine ECG), (2) a 24 h Holter monitoring performed at the time of AF diagnosis, and (3) a complete echocardiographic examination for diagnosis and staging of the underlying cardiac disease.

For each dog included, the following data were collected: signalment, type of structural heart disease associated with AF, disease stage according to the American College of Veterinary Internal Medicine (ACVIM) classification system [13,14], and antiarrhythmic treatments administered.

Dogs were excluded if medical records were incomplete, if either a routine ECG or 24 h Holter monitoring was unavailable, if a diagnosis of lone AF was established, or if antiarrhythmic drugs capable of reducing ventricular premature complexes (VPCs) or the complexity of VAs (e.g., class I, class II, or class III agents [9,10,15]) had been administered between the initial routine ECG and the subsequent Holter monitoring. Conversely, the use of drugs aimed exclusively at controlling mean heart rate (HR) was permitted in dogs with HR > 150 beats/minute at the initial visit and/or mean HR > 125 beats/minute on Holter monitoring [16]. This approach was considered ethically necessary given the negative prognostic impact of uncontrolled HR in dogs with AF [16,17].

### 2.2. Routine ECG and 24 h Holter Monitoring

All electrocardiographic assessments, including routine ECG and 24 h Holter recordings, were interpreted by experienced operators (G.R., C.M., C.V., H.P., C.G.). Standard surface ECGs were acquired at the time of AF diagnosis with a duration ranging from 2 to 5 min. For each dog, the Holter recording selected corresponded to the one closest to the initial ECG and it was required to be performed within 2 weeks of AF diagnosis. The number of days between the ECG and the Holter monitoring was documented for every case.

Routine ECG and Holter recordings were obtained at the authors’ institution using various devices (ECG: Cube ECG, Cardioline S.p.A., Caverano, Italy; TouchECG, Cardioline S.p.A, Trento, Italy; Holter: Cardioline S.p.A., Caverano, Italy; ClickHolter, Cardioline S.p.A, Trento, Italy), following standardized acquisition protocols [8,18]. Holter recordings were performed in the dogs’ home environment during normal daily activities, with owners instructed to maintain a detailed activity log [8]. Data were acquired with 10-bit resolution and a sampling frequency of 250 Hz and subsequently transferred to a computer for analysis. All recordings underwent initial manual review by the operators to evaluate overall quality, verify accurate software detection of each complex, and detect any unrecognized beats [8].

For the purposes of this study, manual differentiation of true VPCs from QRS complex alterations related to rate-dependent aberrancy due to the Ashman phenomenon was considered essential. Differentiation was primarily based on a careful analysis of several electrocardiographic criteria: (1) the coupling interval of the wide QRS complex with the previous beat (true VPCs typically have a fixed coupling interval, while the Ashman phenomenon systematically results from a long R-R/short R-R sequence); (2) the presence of a pause after the wide QRS complex (true VPCs are typically followed by a post-extrasystolic pause, which is usually absent in the Ashman phenomenon); (3) the morphology of the wide QRS complex (the configuration of true VPCs can vary within a recording due to the occurrence of VPCs arising from different ectopic foci, while QRS complex related to the Ashman phenomenon tends to show a stable morphology); and (4) the tendency of the wide QRS complex beats to form groups (VPCs may appear as couplets, triplets, or bigeminy, whereas such type of organization is atypical for the Ashman phenomenon) [8].

After the identification of VPCs, particular emphasis was placed on quantifying and categorizing them as follows: couplets (2 consecutive VPCs), triplets (3 consecutive VPCs), bigeminy (a VPC following every sinus beat), trigeminy (a VPC following every 2 sinus beats), accelerated idioventricular rhythm (≥4 VPCs at a heart rate of 60–180 beats/minute), and ventricular tachycardia (≥4 VPCs at a heart rate >180 beats/minute) [9,10]. Ventricular arrhythmias were classified according to a modified Lown–Wolf grading system: grade 0 = no VPCs; grade 1 = isolated VPCs; grade 2 = ventricular bigeminy or trigeminy; grade 3 = accelerated idioventricular rhythm; grade 4 = ventricular couplets or triplets; and grade 5 = ventricular tachycardia [9,10]. In cases where multiple types of VAs were observed in the same dog, the highest grade of arrhythmia organization was recorded for classification (e.g., if both ventricular bigeminy and tachycardia were observed, the dog was assigned grade 5) [9,10].

### 2.3. Statistical Analysis

Descriptive statistics were performed based on the presence of VPCs detected on the 2 to 5 min ECG recordings, dichotomized into two categories: 0 (absence of VPCs) and ≥1 (presence of VPCs). Categorical variables were expressed as percentages and compared using the chi-square test. Continuous variables were assessed for normality using the Shapiro–Wilk test, reported as mean ± standard deviation, and compared using Student’s *t*-test.

To assess agreement between the 2 min ECG and 24 h Holter monitoring in detecting VPCs, diagnostic test statistics were performed using 2 × 2 contingency tables. Specifically, the presence of ≥1 VPCs on the 2 to 5 min ECG recordings was compared with (1) >100 VPCs/24 h and (2) a Lown–Wolf grade ≥ 4 on Holter monitoring. Considering the Holter recordings as the reference standard and the 2 min ECG as the index test, the following parameters with their corresponding 95% confidence interval (95% CI) were calculated:

- Sensitivity (Se): The probability of a positive ECG result (≥1 VPCs) when the Holter identified severe VAs. Specifically, for the purpose of this study, VAs were classified as severe based on two criteria: (1) the presence of more than 100 VPCs and (2) a Lown–Wolf grade ≥ 4.
- Specificity (Sp): The probability of a negative ECG result (VPCs = 0) when the Holter did not detect severe VAs.
- Prevalence: The proportion of dogs with severe VAs detected by Holter, according to each criterion.
- Positive Predictive Value (PPV): The probability that severe VAs are present when the ECG test is positive:
  PPV = (Se × Prevalence)/[(Se × Prevalence) + ((1 − Sp) × (1 − Prevalence))]
- Negative Predictive Value (NPV): The probability that severe VAs are absent when the ECG test is negative:
  NPV = (Sp × (1 − Prevalence))/[((1 − Se) × Prevalence) + (Sp × (1 − Prevalence))]
- Accuracy: The overall probability that a dog is correctly classified:
  Accuracy = (Se × Prevalence) + (Sp × (1 − Prevalence))

The area under the receiver operating characteristic curve (AUC) was also calculated to evaluate the overall performance of the binary classification. AUC values were interpreted as follows: >0.9 = excellent (highly accurate), 0.8–0.9 = good, 0.7–0.8 = fair, 0.6–0.7 = poor, 0.5 = no discrimination, and <0.5 = worse than random.

To assess the association between the number of VPCs detected on the 2 to 5 min ECG recordings and (1) the total VPC count on Holter and (2) the Lown–Wolf grade, Spearman’s rank correlation coefficient was calculated.

All statistical analyses were performed using MedCalc^®^ Statistical Software version 22.016 (MedCalc Software Ltd., Ostend, Belgium), SAS 9.4 (SAS Institute Inc., Cary, NC, USA), and XLSTAT version 2023.3.0 (https://www.xlstat.com/ accessed on 1 August 2025). For all analyses, a *p*-value < 0.05 was considered statistically significant.

## 3. Results

### 3.1. Animals

A total of 35 dogs with secondary AF met the inclusion criteria, comprising 69% males and 31% females. Most dogs were crossbreds (11 [31.4%]). Purebred dogs included German Shepherd, Golden Retriever and Weimaraner (three [8.6%] dogs each), Corso, Dogue de Bordeaux and Pinscher (two [5.7%] dogs each), and American Bulldog, American Staffordshire Terrier, Appenzeller Sennenhund, Dachshund, Doberman Pinscher, Giant Schnauzer, Leonberger, Rottweiler and Spinone Italiano (one [2.9%] dog each). The mean age was 9.7 ± 3.2 years, and the mean body weight was 31.7 ± 17.5 kg.

Regarding underlying heart disease, 23 (65.7%) dogs had myxomatous mitral valve disease, 9 (25.7%) had DCM, and 3 (8.6%) had congenital heart disease, including 2 cases of tricuspid dysplasia and 1 case of mitral dysplasia. At the time of inclusion, 6 (17.1%) dogs were classified as having compensated heart disease (ACVIM stage B2), while 29 (82.9%) had decompensated disease (27 [93.1%] dogs at ACVIM stage C and 2 [6.9%] dogs at ACVIM stage D).

Table 1 summarizes the selected demographic characteristics and routine ECG findings, including a comparison between dogs with and without VPCs observed on routine ECG.

The median time interval between the acquisition of the routine ECG confirming AF and Holter monitoring was 5 days, with 17 dogs (48.6%) undergoing both tests within 48 h. After the diagnosis of AF, 30 (85.7%) dogs received antiarrhythmic therapy aimed solely at controlling the mean HR. The most commonly prescribed drugs were diltiazem and digoxin. These drugs were administered in combination in 16 (45.7%) dogs, while diltiazem alone and digoxin alone were prescribed in 6 (17.1%) and 8 (22.9%) dogs, respectively.

### 3.2. Electrocardiographic Data

Holter monitoring detected VAs in all dogs, with a range of 3 to 34,531 VPCs (median: 939 VPCs). In contrast, only 13 dogs (37.1%) exhibited recognizable VPCs on routine ECG, with counts ranging from 1 to 10 VPCs (median: 0 VPCs). No significant differences were observed between dogs with and without detectable VPCs on routine ECG in terms of breed, sex, age, body weight, or the type and severity of underlying heart disease. Table 2 and Table 3 summarize selected demographic characteristics, and clinical and ECG findings in dogs with and without VAs on routine ECG, respectively.

On Holter monitoring, 24 (68.6%) dogs had more than 100 VPCs, with a range of 206 to 34531 VPCs (median: 2606 VPCs). Moreover, 27 (77.1%) dogs exhibited VAs with a Lown–Wolf grade ≥ 4. Specifically, 4 (11.4%) dogs showed a grade 1, 3 (8.6%) dogs showed grade 2, 1 (2.9%) dog showed grade 3, 16 (45.7%) dogs showed grade 4, and 11 (31.4%) dogs showed grade 5. All 13 dogs with VPCs on routine ECG had either > 100 VPCs and/or a Lown–Wolf grade ≥ 4 on Holter monitoring.

Spearman’s rank correlation revealed a significant association between the number of VPCs on routine ECG and both the total number of VPCs on Holter monitoring (correlation coefficient = 0.721, *p* < 0.001) and the Lown–Wolf grade (correlation coefficient = 0.411, *p* = 0.016).

The presence of VPCs on routine ECG showed moderate accuracy in predicting severe VAs on Holter monitoring, with AUC values of 0.77 (95% CI: 0.60–0.89) for >100 VPCs and 0.74 (95% CI: 0.57–0.87) for Lown–Wolf grade ≥ 4. Notably, specificity was 100% for both VA severity criteria (95% CI: 71.5–100% for >100 VPCs and 63.1–100% for Lown–Wolf grade ≥ 4), whereas sensitivity was low, ranging from 54.2% (95% CI: 32.8–74.5%) for >100 VPCs to 48.1% (95% CI: 28.7–68.1%) for Lown–Wolf grade ≥ 4.

**Figure 1:**
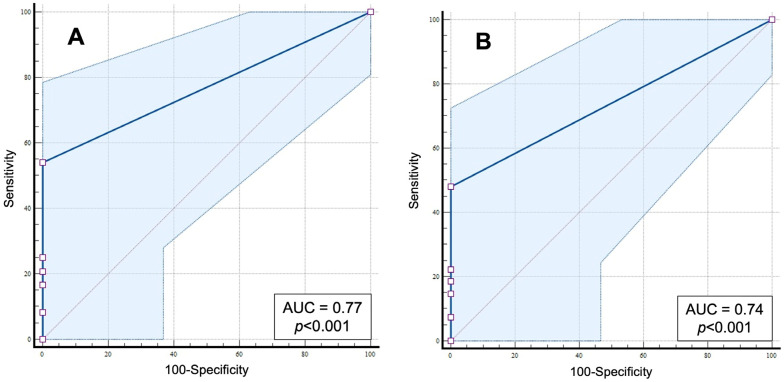
Receiver operating characteristic (ROC) curve of routine electrocardiographic examination for predicting severe ventricular arrhythmias (VAs) on Holter monitoring. (**A**) ROC curve for severity expressed by >100 ventricular complexes on Holter monitoring; (**B**) ROC curve for severity expressed by Lown–Wolf grade ≥ 4 of VAs on Holter monitoring. AUC: area under the ROC curve. The red diagonal line indicates the reference line (AUC = 0.5), representing no discriminatory ability; the blue line indicates the predictive performance with the corresponding 95% confidence interval (light blue area).

Figure 1 and Table 4 present the complete diagnostic accuracy metrics.

## 4. Discussion

This is the first study specifically designed to investigate aspects of the coexistence of VAs and secondary AF in dogs, providing valuable insights not only for a better understanding of this clinical scenario but also for improving the management of similar cases in clinical practice.

First and foremost, this study demonstrated that the presence of VAs is common in dogs with secondary AF. This finding was made possible through the systematic use of Holter monitoring and a meticulous approach to identifying and accurately characterizing VAs. Although this study was not designed to determine the true prevalence of VAs in dogs with AF, it is worth noting that VAs were detected in all dogs diagnosed with secondary AF. Notably, the median number of VPCs recorded during the monitoring period was high (approaching 1000), and the degree of their organization was typically of clinical concern, with a Lown–Wolf grade ≥ 4 observed in more than three-quarters of the study population. These findings underscore the importance of recognizing that, when managing dogs with secondary AF, veterinarians should not focus solely on the medical management of AF (i.e., achieving adequate control of the mean HR [17,19]) and the underlying heart disease (e.g., effective management of congestive heart failure, which often coexists with AF [20]). They should also assess the presence of concomitant VAs and, crucially, evaluate the degree of VPC organization.

Failure to do so may increase the risk of sudden cardiac death, even in dogs with previously well-controlled mean HR (<125 beats/minute [17]) and compensated congestive heart failure, as reported in the veterinary literature [11,19]. Indeed, in dogs with AF, the rate of sudden cardiac death has been reported to be significantly higher compared to that of dogs in sinus rhythm (estimated prevalence of 14.8% and 5.5%, respectively) [11]. It cannot be entirely ruled out that some dogs with AF previously reported to have died of apparently unexplained sudden death may have succumbed to undetected VAs (e.g., when only routine ECG monitoring was performed rather than Holter) or to inadequately treated VAs (e.g., when standard rate control medications were not supplemented with drugs targeting concomitant VPCs organized into complex arrhythmias).

Second, our study highlights that short-duration ECG recordings (2–5 min) are insufficient for the accurate assessment of dogs with secondary AF. Specifically, our statistical analysis demonstrated that brief ECGs lack the sensitivity required to reliably detect severe VAs as identified by Holter monitoring, regardless of the severity criterion applied (i.e., VPC frequency or Lown–Wolf grade).

In other words, while the presence of VPCs on a short ECG may indicate the likelihood of clinically relevant VAs and justify further investigation, their absence does not exclude the presence of significant VAs over the course of the day. Therefore, Holter monitoring remains essential for accurate arrhythmic characterization and informed therapeutic decisions, including the use of antiarrhythmic drugs aimed at controlling VAs. These findings provide a scientific basis to support the recommendation of Holter monitoring, particularly when one or more VPCs are detected during a routine ECG of up to 5 min, even in cases where owners may be hesitant due to cost or logistical concerns. Furthermore, given the low sensitivity of brief ECGs, clinicians should inform owners that declining Holter evaluation may compromise optimal management and increase the risk of undetected VAs, including sudden cardiac death.

Third, it is worth noting that our findings regarding the limitations of short-duration ECG in predicting Holter results align with previous veterinary studies, although these investigations were conducted in different clinical contexts. For example, a study on 431 Doberman Pinschers with DCM and a predominant sinus rhythm compared 5 min ECGs with 24 h Holter monitoring, demonstrating that short ECGs are highly specific but relatively insensitive for detecting VAs. Specifically, a 5 min ECG showing at least one VPC had a specificity of 96.7%, but a sensitivity of only 64.2% for detecting more than 100 VPCs over 24 h [21]. Similar findings were reported in a study of 88 Boxers, where VPCs were evaluated using both a 2–3 min in-hospital ECG and 24 h Holter monitoring [22]. In that study, the ECG was again specific but insensitive for predicting VPCs, with sensitivities of 61% and 76% when the Holter-detected VPC counts were ≥50 and ≥100, respectively [22]. Another investigation assessing the agreement between a non-recordable cage-side continuous ECG and 24 h Holter monitoring in hospitalized dogs with sinus rhythm but at risk for VPCs found only weak concordance between the two methods [23]. Collectively, these studies demonstrate that routine ECG and Holter monitoring are not interchangeable for evaluating VAs in dogs with a dominant sinus rhythm [21,22,23].

Our study extends this concept by showing that the same limitation applies when the dominant cardiac rhythm is AF. Furthermore, a recent study evaluating whether in-clinic ECG-derived HR could predict 24 h Holter-derived mean HR at home found that short ECGs typically overestimate HR, with a median overestimation of 26 beats/minute compared to Holter findings [24]. Clinically, this suggests that a brief ECG is insufficient to determine whether the mean HR in dogs with AF requires pharmacological control, or whether treatment intensification is needed in dogs already receiving rate-controlling medications. Taken together, the existing literature and our findings clearly indicate that managing dogs with secondary AF using only a brief in-clinic ECG is scientifically inadequate and potentially unsafe. In our opinion, the absence of 24 h Holter monitoring in this context compromises both diagnostic accuracy and patient safety, as neither mean HR nor VAs can be reliably assessed and appropriately managed.

This study has some limitations. First, its retrospective design precluded standardization of the timing and duration of diagnostic procedures, including routine ECG recordings, which ranged from 2 to 5 min. Although this variability may have influenced some results, it is important to note that the duration of ECG recordings used in our study reflects common clinical practice, where most veterinarians typically perform in-clinic ECGs for no more than 5 min [21,22,25]. Additionally, some previously published studies on this topic also relied on ECG recordings of variable length [22]. Lastly, it should be considered that, in routine clinical settings, ECG duration is not always easily standardized, for example, uncooperative dogs may require early termination of the recording. Therefore, we believe our findings remain relevant and applicable to everyday veterinary practice.

Second, the criteria used to define severe VAs should be carefully considered when interpreting our results. We adopted two parameters: one based on VPC frequency (>100 VPCs over 24 h) and the other on their degree of organization (Lown–Wolf grade ≥ 4). The >100 VPCs threshold was selected because studies have shown that, while occasional VPCs can occur in healthy adult dogs, exceeding 100 VPCs in 24 h is uncommon [26,27,28,29]. Consequently, identifying more than 100 VPCs during Holter monitoring likely represents a non-random, clinically relevant arrhythmia warranting attention [21,24]. For the second criterion, we focused on VAs reaching at least grade 4 in the Lown–Wolf classification. In human medicine, higher Lown–Wolf grades are associated with increased risk of cardiac death, particularly in post-myocardial infarction patients [30]. Similarly, in dogs, grade 5 VAs (i.e., ventricular tachycardia) can degenerate into ventricular fibrillation, causing sudden death [12], and even grade 4 VAs (i.e., couplets and triplets) carry negative prognostic significance [7]. Using only one of these criteria would have been insufficient for a clinically meaningful comparison between routine ECG and Holter findings. The cut-offs we selected are therefore supported by scientific evidence and are likely to have practical relevance in veterinary practice. However, different results might be obtained if alternative Holter cut-offs were applied.

Third, the study population was relatively small and heterogeneous regarding both breed and underlying cardiac disease. Nevertheless, assembling a study sample in which all dogs undergo Holter monitoring is inherently challenging. This can be evidenced by the fact that other studies using Holter monitoring as an inclusion criterion often enrolled a comparable number of dogs [8,24,29]. Importantly, including multiple breeds and various cardiac conditions enhances the generalizability of our findings to real-world veterinary practice, where dogs of diverse breeds and cardiac profiles are commonly evaluated. In contrast, limiting the study to a single breed or condition (e.g., only Doberman Pinschers with DCM) would have restricted clinical applicability.

Fourth, routine ECG and Holter monitoring were not always performed on the same day, with a median interval of 5 days between tests. While this time gap could potentially represent a source of bias, in clinical practice it is often not feasible to perform Holter monitoring on the same day that AF is diagnosed. This limitation has also been noted in previous studies involving dogs with AF [11,16], and the median interval observed here aligns with those reported in prior investigations of dogs with this tachyarrhythmia [11].

Fifth, although digoxin is routinely used for rate control in dogs with AF, it has proarrhythmic potential. Indeed, its action increases intracellular calcium concentration in cardiomyocytes, which enhances contractility but also facilitates delayed afterdepolarizations and triggered activity, thereby predisposing to VAs [31,32,33]. Therefore, it cannot be conclusively ruled out that part of our findings may have been influenced by the administration of this drug.

Another limitation is the absence of data on the rate of collapse or transient loss of consciousness in the included dogs, documented before and after the initiation of antiarrhythmic therapy. Similarly, no statistical analysis was performed to assess whether dogs with more severe VAs on Holter monitoring had a significantly higher risk of experiencing collapse or transient loss of consciousness.

Lastly, dogs underwent 24 h Holter monitoring rather than longer recordings. Extended monitoring, ideally lasting at least 3 days, could enhance the detection of ventricular arrhythmias [34]. Additionally, in a 24 h Holter, the total daily number of VPCs may be influenced by the spontaneous variability of ventricular ectopy, which can reach up to 80% [35]. Nevertheless, in routine clinical practice, Holter recordings longer than 24 h are often impractical due to higher costs and limited tolerance by the dogs.

## 5. Conclusions

In conclusion, our study demonstrates that brief in-hospital ECG recordings have high specificity but low sensitivity for detecting VAs in dogs with secondary AF. The presence of VPCs on a short ECG should prompt further investigation by Holter monitoring, primarily to better characterize their frequency and complexity and to guide the need for targeted antiarrhythmic therapy. However, the absence of VPCs on a 2–5 min ECG does not exclude the presence of VAs, including clinically relevant arrhythmias such as ventricular tachycardia, at other times during the day. Further research is warranted to clarify the potential prognostic significance of VAs in dogs with secondary AF.

## Figures and Tables

**Table 1:** Selected demographic characteristics of 35 dogs with secondary atrial fibrillation and ventricular premature complexes (VPCs) confirmed by 24 h Holter monitoring, with comparison between dogs with and without VPCs detected on routine ECG.

Variable	All Dogs	VPCs on ECG		*p*-Value
		Yes	No	
N. of dogs (%)	35	13 (37)	22 (63)	-
Breed: CB, PB (%)	11 (31), 24 (69)	5 (38), 8 (62)	6 (27), 16 (73)	0.76
Sex: M, F (%)	24 (69), 11 (31)	7 (54), 6 (46)	17 (77), 5 (23)	0.29
Age (y)	9.7 ± 3.2	10.2 ± 3.0	9.4 ± 3.4	0.50
Weight (kg)	31.7 ± 17.5	33.4 ± 16.5	30.8 ± 18.3	0.68
HD: MMVD, DCM, CHD (%)	23 (66), 9 (26), 3 (8)	10 (77), 3 (23), 0 (0)	13 (59), 6 (27), 3 (14)	>0.30
ACVIM stage: B2, C + D (%)	6 (17), 29 (83)	2 (15), 11 (85)	4 (18), 18 (82)	0.99

ACVIM: American College of Veterinary Internal Medicine; CB: crossbred; CHD: congenital heart disease; DCM: dilated cardiomyopathy; F: female; HD: heart disease; M: male; MMVD: myxomatous mitral valve disease; PB: purebred.

**Table 2:** Selected demographic characteristics and clinical and electrocardiographic findings in 13 dogs with secondary atrial fibrillation and recognizable ventricular arrhythmias on routine electrocardiographic examination.

Breed	Sex	Age (Year)	BW (kg)	Cardiac Disease	ACVIM Stage	Number of VPCs/ECG	Lown–Wolf Grade/ECG	Number of VPCs/Holter	Lown–Wolf Grade/Holter
Corso	IF	9	72	MMVD	C	1	1	3381	5
Crossbred	SF	9	29	MMVD	C	1	1	3580	4
German Shepherd	SF	13	41.5	MMVD	B2	1	1	2606	4
Crossbred	IM	15	27.5	MMVD	C	1	1	3388	4
Crossbred	SF	12	11.2	MMVD	D	1	1	206	5
Dogue de Bordeaux	IF	3	52.5	DCM	C	5	1	12,151	4
Doberman Pinscher	IM	10	33.8	DCM	D	1	1	3436	4
Weimaraner	CM	12	31.5	MMVD	C	4	2	1417	4
Appenzeller Sennehund	IM	11	24.5	MMVD	C	9	2	8926	5
Giant Schnauzer	SF	12	31.1	MMVD	B2	9	2	34,531	4
Golden Retriever	IM	7	30	MMVD	C	1	1	5572	5
Crossbred	CM	9	41	DCM	C	10	2	12,099	5
Crossbred	CM	11	8.2	MMVD	C	10	4	2640	5

ACVIM: American College of Veterinary Internal Medicine; BW: bodyweight; CM: castrated male; DCM: dilated cardiomyopathy; ECG: routine electrocardiogram; IF: intact female; IM: intact male; MMVD: myxomatous mitral valve disease; SF: spayed female; VPC: ventricular premature complex.

**Table 3:** Selected demographic characteristics and clinical and electrocardiographic findings in 22 dogs with secondary atrial fibrillation but without recognizable ventricular arrhythmias on routine electrocardiographic examination.

Breed	Sex	Age (Year)	BW (kg)	Cardiac Disease	ACVIM Stage	Number of VPCs/Holter	Lown–Wolf Class/Holter
Dogue de Bordeaux	CM	7	56.5	DCM	B2	72	5
Leonberger	IM	6	62	DCM	C	1733	4
Golden Retriever	IM	11	34.5	MMVD	C	1024	5
Italian Spinone	CM	14	18.5	MMVD	C	4	1
Corso	IM	8	73	DCM	C	9	1
Miniature Pinscher	SF	9	3.4	MMVD	C	93	4
American Staffordshire Terrier	SF	9	22.5	MD	C	329	5
German Shepherd	IM	7	33	MMVD	C	86	2
Weimaraner	SF	11	29	MMVD	C	41	2
Crossbred	CM	10	29.3	DCM	C	719	4
Crossbred	CM	11	15.4	MMVD	C	2000	5
Crossbred	SF	9	46.1	MMVD	C	939	4
Crossbred	CM	13	30	MMVD	C	585	4
Pinscher	IM	11	5.9	MMVD	C	3	4
Dachshund	IM	9	7.5	MMVD	C	33	1
German Shepherd	IF	11	34	MMVD	C	1283	4
Crossbred	CM	13	16.5	MMVD	C	8638	4
American Bulldog	IM	7	46	DCM	C	15	2
Crossbred	CM	14	8.2	MMVD	C	355	3
Weimaraner	IM	2	35	TD	B2	24	4
Rottweiler	IM	2	37.2	TD	B2	324	5
Golden Retriever	IM	14	34	DCM	B2	32	1

ACVIM: American College of Veterinary Internal Medicine; BW: bodyweight; CM: castrated male; DCM: dilated cardiomyopathy; ECG: routine electrocardiogram; IF: intact female; IM: intact male; MD: mitral dysplasia; MMVD: myxomatous mitral valve disease; SF: spayed female; TD: tricuspid dysplasia; VPC: ventricular premature complex.

**Table 4:** Diagnostic accuracy of >1 ventricular premature complex (VPC) detected by routine ECG in predicting severe ventricular arrhythmia (VA) on 24 h Holter monitoring.

VA Severity	Number of Dogs (%)	Sensitivity (95% CI)	Specificity (95% CI)	AUC (95% CI)	PPV (95% CI)	NPV (95% CI)	Accuracy (95% CI)
>100 VPCs on Holter	24/35 (69%)	54.2 (32.8–74.5)	100 (71.5–100)	0.77 (0.60–0.89)	100 (75.3–100)	50 (39.3–60.7)	68.6 (51.7–83.1)
Lown–Wolf grade ≥ 4	27/35 (77%)	48.1 (28.7–68.1)	100 (63.1–100)	0.74 (0.57–0.87)	100 (75.3–100)	36.4 (28.4–45.1)	60 (42.1–76.1)

AUC: area under the curve; CI: confidence interval; NPV: negative predictive value; PPV: positive predictive value.

## Data Availability

All the data are available in the present manuscript.
